# The social dynamics of complex gestural communication in great and lesser apes (*Pan troglodytes*, *Pongo abelii, Symphalangus syndactylus*)

**DOI:** 10.1098/rstb.2021.0299

**Published:** 2022-09-26

**Authors:** Federica Amici, Katja Liebal

**Affiliations:** ^1^ Leipzig University, Life Sciences, Institute of Biology, 04103 Leipzig, Germany; ^2^ Department of Human Behavior, Ecology and Culture, Max Planck Institute for Evolutionary Anthropology, 04103 Leipzig, Germany

**Keywords:** sociality, chimpanzees, siamangs, orangutans, complex gesture use

## Abstract

Gestures play an essential role in primate communication. However, little is known about how complexity of gestural use (in terms of repertoire size, intentional use, flexibility and use of gestural sequences) relates to individual and dyadic measures of sociality and whether more complex gestural use is more effective in eliciting a response. We observed 19 captive chimpanzees (*Pan troglodytes*), 16 Sumatran orangutans (*Pongo abelii*) and 18 siamangs (*Symphalangus syndactylus*) to assess the complexity and effectiveness of their gestural use. We found that, beyond interspecies variation, the number of gesture types used in a dyad was higher when individuals had stronger social bonds; the probability of accounting for others' attention increased with age, especially for visual gestures; and sequences were more likely used by younger or socially less integrated individuals. In terms of effectiveness, older individuals and those using fewer sequences were more likely to be responded to, while across dyads, the probability of obtaining a response was higher when both individuals accounted for the other's attention and when they used fewer sequences. Overall, this confirms the link between sociality and complex gestural use and suggests that more complex forms of communication, at least in terms of intentional use, may be more effective at achieving communicative goals.

This article is part of the theme issue ‘Cognition, communication and social bonds in primates’.

## 1. Introduction

In the past decades, gestural communication in non-human primates (hereafter, primates) has been the focus of abundant research. By using observational and experimental methods in wild and captive settings, researchers have started revealing the complexity of primate gestural communication, and the flexible way in which gestures can be used by different species [1,2]. To date, we know that gestures play an essential role in primate communication systems of both monkeys and apes [3–5]. Moreover, gestures’ defining features (e.g. open-ended repertoires, intentional and flexible use) resemble some of the ‘design features’ of human language [6], and several researchers have proposed a crucial role of gestures for the evolution of human language [7–10].

So far, researchers have identified several hallmarks of complexity in gestural communication systems, including repertoire size, intentional use, flexibility and use of sequences of gestures ([10]; see [11,12]). The size of a species repertoire is usually defined as the number of different gesture types produced by individuals across conspecific groups [13]. Therefore, repertoire size is highly dependent on the way gestures are operationally defined, and in particular on how fine-graded distinctions between different gestural categories are (see [14]). In chimpanzees (*Pan troglodytes*), for instance, the species repertoire size is quite large, but it can vary from less than 30 [15] to more than one hundred gestures [16], depending on the methodological approach used to identify gesture types. Moreover, repertoire size may substantially vary across individuals of the same species, as not all individuals necessarily display the whole species-specific gestural repertoire. In chimpanzees, for instance, individual repertoire size includes on average only around 10 gestures, suggesting high intra-species variation in the number and types of gestures that individuals produce [15]. Repertoire size also varies with primate age, with the first gestures emerging around 8–12 months in great apes [17], repertoire size reaching a peak in juveniles and gradually decreasing through adulthood [14].

A second important hallmark of complexity in gestural communication is intentional use [2,10]. When intentionally communicating with others, individuals may have to use complex cognitive skills to reach their communicative goals, by for instance adjusting to the recipients' attentional states or persisting in their signalling until obtaining a response [2,16]. For visual signals to work, for instance, recipients need to be visually attending to the signaller, and the signaller may account for the receiver's attention before gesturing [18]. To date, there is no consensus on how intentional use should be operationalized, and researchers often rely on different criteria to assess its occurrence during gestural production (see [10,19]). Primates, for instance, are considered to intentionally use gestures when these (i) are produced in the presence of other individuals, (ii) are directed toward attentive recipients (especially in the case of visual gestures, which can only be perceived by recipients that are visually attentive to the signal) and/or (iii) are persistently produced until they elicit a response [1,3,20,21]. According to the criteria above, several primate species appear to produce gestures intentionally [1,10], although there may be significant variation across species and individuals [2,10]. In chimpanzees, for instance, intentional instances of communication appear to increase with age, but they also vary depending on the identity of the recipients, being less frequent when partners are strongly related (e.g. mothers) and the outcome of their interactions is more predictable [22].

Another hallmark of complexity in gestural communication is flexibility, which has been defined as the use of a specific gesture type in many different contexts, or as the use of many different gesture types in a certain context [16,23,24]. In primates, gestures may be used in a flexible way (see e.g. [7]), although gesture types clearly vary with regard to their context specificity and to their strength of association to specific goals [24]. To date, several primate species have been shown to use gestures flexibly. In captive orangutans, for instance, the largest majority of gestures are used flexibly in more than one functional context, and many different signals can be used within the same context [25]. Similarly, wild chimpanzees have been shown to use gesture types in several contexts, with some gestures being used in up to nine different contexts [14].

Finally, primates may combine gestures into longer sequences to better achieve their communicative goals. Primates, for instance, may combine different gestures or persistently repeat the same gesture to elicit a response [26,27]. However, it is not clear whether the use of gestural sequences really reflects gestural complexity. For instance, there is to date no clear evidence that primates can combine gestures into longer sequences that have a novel meaning [28–32]. In chimpanzees, gestural sequences are common, but they are mostly redundant repetitions of the same gesture types [28,29]. Similarly, gestural sequences in orangutans largely consist of repetitions of the same gestures, and may persist also after the recipient responds, suggesting that they are largely emotionally based [32]. Furthermore, gestural sequences appear to become less frequent as individuals get older [28], suggesting that gestural sequences, after all, may not reflect gestural complexity, but that they are rather used by inexperienced and/or emotionally aroused individuals after failed communicative attempts.

In this study, we compared the gestural repertoires of several ape species (i.e. chimpanzees, *Pan troglodytes*, Sumatran orangutans, *Pongo abelii,* and siamangs, *Symphalangus syndactylus*) to assess (i) how complexity of gestural use (in terms of repertoire size, intentional use, flexibility and use of gestural sequences) relates to individual and dyadic measures of sociality and (ii) whether complex gestural use is more effective at achieving the communication goals (i.e. eliciting the recipient's response). First, we assessed whether complex gestural communication is predicted by individual differences in social experience (i.e. integration in the social network) and dyadic measures of relationship quality (i.e. maternal kin, social bonds). In particular, we hypothesized that, if social experience has an important role in shaping individual gestural communication, higher integration in the social network and better relationship quality might provide individuals with more opportunities to interact with others, practice and refine their communicative skills. Therefore, we predicted that more integrated individuals and dyads with better relationship quality would show more complex gestural use (i.e. larger repertoire sizes, higher probability of taking into account recipients' attentional states when producing visual gestures, higher flexibility in gestural use and higher frequency of gestural sequences; for a list of predictions, see table 1). As social experience increases with age, we also predicted that older individuals would show more complex gestural use. Second, we assessed whether complex gestural communication is more effective at eliciting recipients’ responses. In particular, we predicted that the probability of eliciting recipients' response would be higher when individuals and dyads use more complex forms of gestural communication. Moreover, we predicted that the probability of eliciting a response would change depending on social experience, being higher for more integrated and older individuals, and in dyads with better relationship quality (table 1). Finally, given that our study sample included different species, we also explored interspecies differences in the complexity of gestural communication and in the effectiveness of their communication systems. As our study sample only included three species that differ in several socio-ecological aspects potentially linked to complex communication (e.g. fission–fusion levels [33]; dominance style [34,35]), these analyses are only exploratory and the results will only be interpreted *post-hoc* in the discussion.

**Table 1.** For each level of our analysis, detailed predictions, models used to test them, and whether our predictions were confirmed. (For model definitions, see §2d.)

predictions	model	confirmed?
At the individual level, more social integration (i.e. centrality) and older age predict more gestural complexity, in terms of:		
> repertoire size	M1-Ind	no
> probability of accounting for others’ attentional states	M2-Ind	yes (age)
> flexibility	M3-Ind	no
> probability of using gestural sequences	M4-Ind	no (<)
At the dyadic level, better relationship quality (i.e. kin, bonds) predicts more gestural complexity, in terms of:		
> repertoire size	M1-Dyad	yes (bonds)
> probability of accounting for others’ attentional states	M2-Dyad	no
> flexibility	M3-Dyad	no
> probability of using gestural sequences	M4-Dyad	no
At the individual level, more effective communication is predicted by:		
> gestural complexity (i.e. > repertoire size, > probability of accounting for others’ attentional states, > flexibility, > probability of using gestural sequences)	M5-Ind	no (< sequences)
> social integration, > age	M5-Ind	yes (age)
At the dyadic level, more effective communication is predicted by:		
> gestural complexity (i.e. > repertoire size, > probability of accounting for others’ attentional states, > flexibility, > probability of using gestural sequences)	M5-Dyad	yes/no (attention/< sequences)
> kin, > bonds	M5-Dyad	no

## 2. Methods

### (a) Study subjects

Fifty-three captive apes participated in our study (see electronic supplementary material, table S1 for more details). First, we included four groups of siamangs (*N* = 18), two family groups (both *N* = 4) housed at the Zoo Krefeld (Germany) and two family groups (both *N* = 5) at the Howletts Wild Animal Park in Bekesbourne (United Kingdom). All groups lived in large external enclosures with access to adjacent sleeping rooms, except for Group 1 at Zoo Krefeld, which lived in an indoor enclosure where it was possible to observe the group during the whole day. Second, we included two groups of Sumatran orangutans (*N* = 16), one (*N* = 9) housed at the Zürich Zoo (Switzerland) and one (*N* = 7) at the Leipzig Zoo (Germany). Both groups were housed in an indoor and outdoor enclosure containing several trees, ropes and platforms. Finally, we included one group of chimpanzees (*N* = 19) at the Yerkes Regional Primate Research Center (Field Station) in Atlanta, Georgia, USA. The group lived in an outdoor enclosure with a wooden structure in the centre and various objects (e.g. toys, barrels, branches) throughout the compound, and an adjacent indoor enclosure with sleeping and study rooms.

According to the STRANGE framework [36], our study sample appears to be relatively representative: (i) our study subjects included individuals living in social groups, with different social ranks, that were observed in their social groups; (ii) there were no systematic biases in participation, as all individuals in the study groups were observed (except for one siamang group, see below); (iii) all subjects but three were born in captivity, but they lived in social groups and experienced regular enrichment activities when the observations took place; (iv) all subjects were well habituated to the presence of human observers; (v) we specifically accounted for differences in individual developmental stages; (vi) study subjects were captive, but they did not belong to a specific genetic line and included both males and females; and (vii) several study subjects had participated in different behavioural and cognitive experiments, but we consider it unlikely that these previous experiments might have had long-term effects on the natural occurrence of gestural communication in these groups.

### (b) Data collection

For both siamangs and orangutans, we used 15-minute bouts of focal-animal sampling [37] to observe each individual of the group, for a total of 10 h per individual (except for group B at Howletts Wild Animal Park, where only the youngest individual was followed with focal-animal sampling). Every focal animal was selected in a random order and was videotaped in 15-min bouts. If a subject moved outside the range of vision the recording was stopped, and if it did not return within 5 min the next session with a new focal animal was started. Daily observations took place between 7.30 am and 6 pm on every week day, with observation times equally distributed between mornings and afternoons.

For chimpanzees, unlike for the other species, focal animal sampling consisted of 5-minute bouts. Most observations were conducted in the mornings from 8 to 12 am, three to four times per week, but other observations were also conducted in the afternoon. For each session, each individual was randomly selected and videotaped for 5 min, and once this time had elapsed the next subject was selected until all subjects had been followed once. The total observation time amounted to 42 h of focal-animal sampling, for an average of more than 2 h of observation per individual. This resulted in a comparable number of gestures being observed in all species (i.e. mean number of gestures observed for each individual, *N* = 63 in chimpanzees, *N* = 59 in orangutans, *N* = 80 in siamangs). For more details on the observational effort, please see electronic supplementary material, table S2. All observations were video-recorded and later coded for analyses. Part of the data analysed here has already been used in other studies to address different research questions [25,29,38].

### (c) Coding

**Figure 1.**
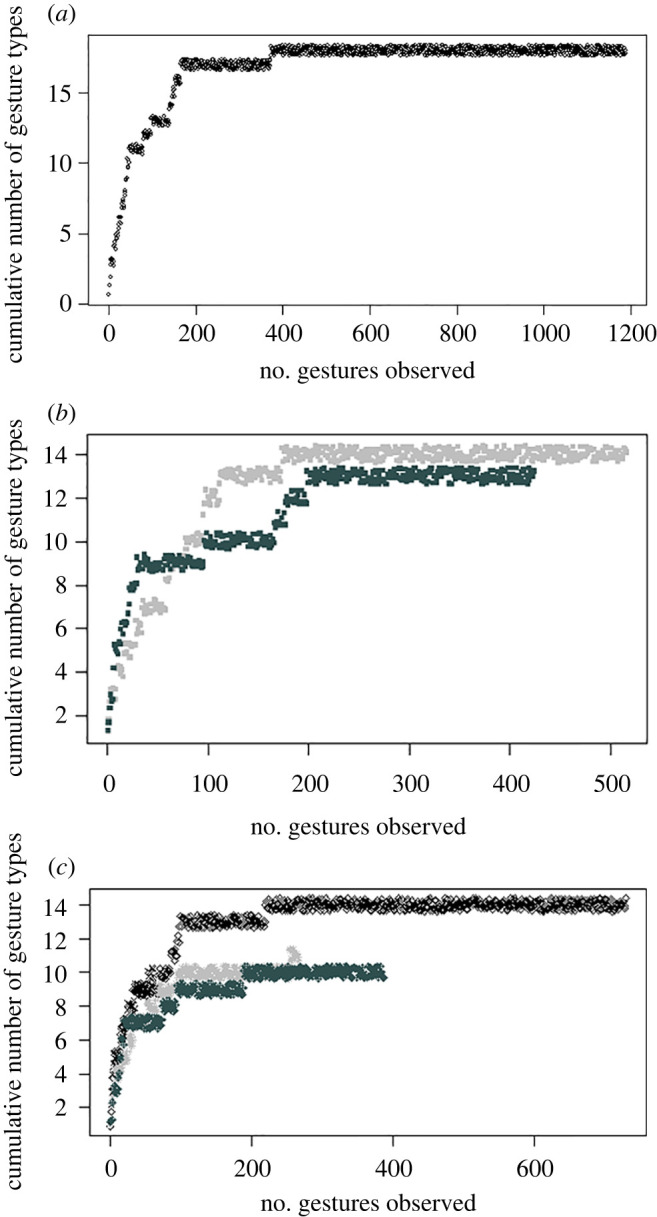
Cumulative number of gesture types observed in the study groups, as a function of the number of gestures coded, separately for each species ((*a*) chimpanzees, (*b*) orangutans, (*c*) siamangs) and group (circles: chimpanzees in Atlanta; light and dark grey squares: orangutans at the Leipzig and Zürich Zoos, respectively; black crosses and black diamonds: siamangs at the Howletts Wild Animal Park, groups A and B, respectively; light grey asterisks and dark grey diamonds: siamangs at the Krefeld Zoo, groups 1 and 2, respectively).

From the videos, we extracted two kinds of information, i.e. on gestural communication and on the social relationships across group members. To acquire information on ape gestural communication, we used Adobe Premiere and VLC media player to code all visible instances of gestures involving the focal subject. We operationally defined gestures as all expressive movements of head or limbs and body postures (excluding whole-body actions) that were directed toward a particular recipient and showed some sign of flexible use (e.g. response-waiting, persistence, means-end dissociation [18,27,39]). To differentiate gestures types, we used previously established categorizations of gestural types already reported in literature [25,29,38] but removed whole-body actions. Our approach did not prioritize fine-graded distinctions between versions of different gestures (e.g. we did not differentiate between gestures performed with one hand versus two hands). Consequently, we had relatively few gesture types for each species (cf. [16]) and asymptote could be reached with lower observational effort (figure 1). Our final list of gestures included 18 gesture types for chimpanzees, 17 for orangutans and 14 for siamangs (see electronic supplementary material, table S3).

For every recorded signal, we coded: (i) gesture type and modality (i.e. visual or non-visual); (ii) sender and recipient identity; (iii) recipient's attentional state (i.e. whether the recipient had direct eye contact with the sender, or the body oriented toward the sender and the sender in his field of vision, and his attention was not distracted by other social partners or happenings in the environment); (iv) recipient's response (i.e. whether there was a reaction by the recipient within a time interval of 5 s from the gesture, including agonistic, affiliative or sexual responses and production of other signals); (v) functional context in which the gesture was produced (e.g. agonistic, affiliative, sexual, playful); and (vi) whether the gesture produced was part of a sequence (i.e. if gestures were produced by the same individual toward the same recipient within 5 s, in the same context; see e.g. [29]). Inter-observer reliability for this dataset was assessed in previous publications [25,29,38] by a second person re-coding 20% of the data, and it was good (Cohen's kappa for gesture type in chimpanzees: 0.69; in orangutans: 0.77; in siamangs: 0.71; for modality in chimpanzees: 0.71; for attentional state in chimpanzees: 0.75; for recipient's response in chimpanzees: 0.79).

To acquire information on the social relationships across group members, we conducted scans [37] from the videos, recording all individuals within 2 m from the focal subject (except for the group B at Howletts Wild Animal Park, where the scan was conducted on the first visible individual from a pseudo-randomized list). We only conducted scans when the video allowed coding in the 2 m range from the focal subject. Although differences in social relationships should be ideally assessed with composite indexes including several affiliative measures (e.g. grooming, proximity; see e.g. [40]), we unfortunately did not have enough data for all groups and species to use a different approach. We conducted up to one scan every 10 min, resulting in a total of 35 scans for each study subject, except for group A at Howletts Wild Animal Park (where we conducted 10 scans for each study subject) and for the chimpanzees at the Yerkes Regional Primate Research Center (where we conducted a total of 163 scans over 5-minute bouts, i.e. on average 7 scans, ranging from 4 to 10 for each individual). We then used these proximity measures to build an undirected weighted matrix for each group, and to obtain the group social networks and the individual Eigenvector centralities (i.e. the sum of the centralities of an individual's neighbours, a measure of the importance of each individual ‘as a social hub’ [41,42]). For this purpose, we used the following packages in R [43]: vegan (v. 2.5–3 [44]), asnipe (v. 1.1.10 [45]) and igraph (v. 1.2.1 [46]). Social network analyses revealed the existence of several communities (2–4) within all chimpanzee and orangutan groups, but only one in each siamang group, likely reflecting the fission–fusion dynamics that characterize the former species in the wild. Individual centralities were assessed within each group separately, and could vary from 0 to 1, with 0 being assigned to the least socially integrated individuals [41,42]. Finally, we used proximity measures to assess the quality of dyadic relationships within each study group. Following Silk and colleagues [40], for each dyad we calculated the ratio between the number of observations in which the two individuals were in proximity and the total number of observations of the two individuals. We then averaged this value for the group and divided all the dyadic ratios for the group average, obtaining dyadic scores that could vary between zero and infinity, with values below 1 representing weaker than average social relationships and values above 1 representing stronger than average ones [44].

### (d) Statistical analyses

First, we plotted the cumulative number of gesture types observed in each study group on the number of gestures observed, to visually assess whether gestural repertoires reached an asymptote (figure 1). The figure suggests that asymptote was reached after approximately 200–400 gestures for all study groups. We then used generalized linear mixed models [47] with the brms package (v. 2.16.3 [48]) in R [43]. We first ran four sets of models to assess whether more integrated and older individuals showed more complex gestural use (table 1) and explored interspecies variation in gestural complexity. In each of the four sets of models, we used one of the following responses (as measures of gestural complexity): individual repertoire size (modelled with a binomial distribution, as the number of gesture types produced by each individual out of the total number of gesture types produced by each species observed: M1-Ind); individual probability of accounting for recipients' attentional states (modelled with a binomial distribution, as the number of gestures accounting for the recipients’ attentional state out of the total number of gestures for which this could be assessed: M2-Ind); individual flexibility (calculated by first identifying, for each individual and gesture type produced at least twice, the most common context in which it was produced, then assessing the individual proportion of gestures of that type that was produced in that context, and finally averaging the scores for each individual across gesture types [16] and modelling these proportions with a beta distribution: M3-Ind); and individual probability of using gestural sequences (modelled with a binomial distribution, as the number of gestures that were part of a sequence out of the total number of gestures for which we could assess it: M4-Ind). In all these sets of models, we included individuals' centrality, age and species as main predictors. In M2-Ind, these predictors were also included in three 2-way interactions with gesture modality (i.e. visual or non-visual), as individuals were expected to account for recipients’ attentional states more/only in the visual modality. In all sets of models, we controlled for sex. We also controlled for observational effort (i.e. number of gestures observed in the subject), but not in M2-Ind and M4-Ind, as observational effort was already largely entailed in the response (as the total number of gestures for which we could assess recipient's attentional state/whether they were part of a sequence). As we only entered one line per individual (*N* = 53), we included no random factors in the models, except for M2-Ind, where we entered one line for each individual and modality (*N* = 106) and included individual identity as random factor. For each set of models, we finally compared the models above to simpler models only including random factors and controls (i.e. observational effort and sex in M1-Ind and M3-Ind, sex in M2-Ind and M4-Ind) and for M3-Ind to an intermediate model in which the three 2-way interactions were removed and only the main terms were included as predictors.

We then ran four similar sets of models at the dyadic level, to assess whether dyads with better relationship quality showed more complex gestural use (table 1). We used similar responses for our models (but assessed at the dyadic level) and identical distributions as for the individual models: dyadic repertoire size (M1-Dyad), dyadic probability of accounting for recipients' attentional states (M2-Dyad); dyadic flexibility (M3-Dyad) and dyadic probability of using gestural sequences (M4-Dyad). In all these sets of models, we included maternal kinship, dyadic bond and species as main predictors. We always controlled for absolute age difference and sex combination, and as above, in M1-Dyad and M3-Dyad, for observational effort. As each individual was included in multiple dyads (*N* = 210), we entered both individual identities as random factors in all the dyadic models, using multi-membership models to account for the fact that the same individual identities can appear in both variables (individual 1 or individual 2 in each dyad) and thus control for the lack of independency in these data points. For each set of models, we finally compared the models above to simpler models only including random factors and controls (i.e. observational effort, age difference and sex combination in M1-Dyad and M3-Dyad, age difference and sex combination in M2-Dyad and M4-Dyad).

Finally, we ran two last sets of models to assess whether the probability of eliciting recipients’ response was predicted by complex gestural use and social experience. At the individual level, we assessed whether the probability that individuals elicited a response (modelled with a binomial distribution, as the number of gestures that were responded to out of the number of gestures produced: M5-Ind) was predicted by individual repertoire size (as operationalized in M1-Ind), individual probability of accounting for recipients' attentional states (as in M2-Ind, but only for gestures in the visual modality), individual flexibility (as in M3-Ind) and individual probability of using gestural sequences (as in M4-Ind). We further included individuals’ centrality, age and species as main predictors, and controlled for sex. As we only entered one line per individual (*N* = 53), we included no random factors in this set of models. At the dyadic level, we assessed whether the probability of eliciting a response in the dyad was predicted by dyadic repertoire size (as operationalized in M1-Dyad), dyadic probability of accounting for recipients' attentional states (as in M2-Dyad), dyadic flexibility (as in M3-Dyad) and dyadic probability of using gestural sequences (as in M4-Dyad). We further included maternal kinship, dyadic bond and species as main predictors, and controlled for absolute age difference and sex combination. As each individual was included in multiple dyads (*N* = 210), we entered both individual identities as random factors, as for the other dyadic models. For both sets of models, we then compared these models to simpler ones only including random factors and controls (i.e. sex in M5-Ind, age difference and sex combination in M5-Dyad) and to intermediate models that, compared to the simpler models, also included measures of sociality (i.e. centrality, age and species in M5-Ind; maternal kinship, dyadic bond and species in M5-Dyad).

In all models, we *z*-transformed continuous predictors and controls (i.e. centrality, age, dyadic bond, age difference, observational effort). Models were compared using the approximate leave-one-out (loo) cross-validation in the loo package [49] and selecting the best model based on the difference (and standard error) between the expected log pointwise predictive densities (elpd) of the full and null models [50]. All models were run using flat priors, four chains in parallel (to increase the number of independent samples from our models and improve inference accuracy) with 2000 iterations each, half of which were warm-up samples (to improve sampling efficiency [51]). For categorical predictors (i.e. species), we used the emmeans package (v. 1.5.0 [52]) to conduct *post-hoc* comparisons. We conducted posterior predictive checks using the bayesplot package [53]. Convergence was suggested by a high effective number of samples in our models and Rhat estimates of 1.00 [51]. We found no collinearity issues in the models presented (maximum variance inflation factors = 2.41).

## 3. Results

### (a) Individual social integration and gestural complexity

**Figure 2.**
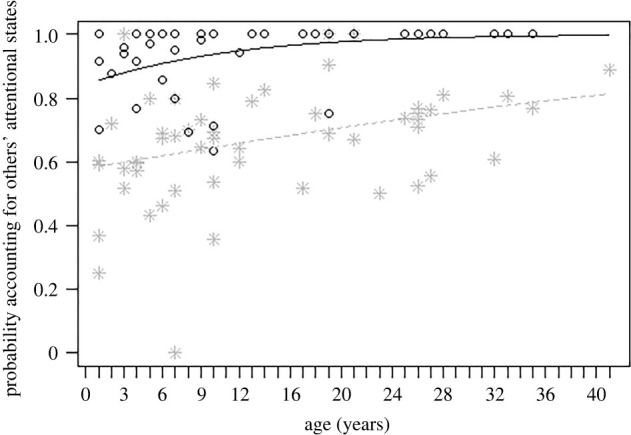
For all species, individual proportion of gestures in which the individual accounted for the recipients’ attentional state when gestures were produced in the visual or in the non-visual modality, as a function of the individual's age (in years). Circles represent individual proportions in the visual modality, and grey asterisks in the non-visual modality. The two lines represent the fitted model, which is like Model M2-Ind, but unconditional on the other predictors that were standardized.

For M1-Ind, the simpler model provided a slightly better fit to the data than the more complex one (elpd difference: 1.0 ± 2.2), suggesting that repertoire size was not predicted by social integration, age or species. For M2-Ind, the most complex model fit the data better than the simplest one (elpd difference: −54.0 ± 29.3) and partially better than the intermediate one (elpd difference: −4.1 ± 5.8). In particular, the probability of accounting for recipients’ attentional states generally increased with age (*β* = 0.30, lower-upper 95% CIs = 0.13 to 0.46), especially in the visual modality (*β* = 1.06, lower-upper 95% CIs = 0.30 to 2.03; figure 2). Moreover, although all species accounted for others' attentional states more in the visual than in the non-visual modality, there were differences across species: orangutans (probability = 0.996, lower-upper 0.95 HPD = 0.986 to 1.000) scored higher than siamangs (probability = 0.968, lower-upper 0.95 HPD = 0.929 to 0.993), which in turned scored higher than chimpanzees (probability = 0.944, lower-upper 0.95 HPD = 0.894 to 0.984) in the visual modality. For M3-Ind, in contrast, the more complex model provided a slightly better fit to the data than the simpler one, but the difference was minimal (elpd difference: −0.6 ± 2.9), suggesting that gestural flexibility was not reliably predicted by social integration, age or species. Finally, for M4-Ind, the more complex model provided a better fit to the data than the simpler one (elpd difference: −34.9 ± 27.4). In particular, the probability of using gestural sequences decreased with age (*β* = −0.29, lower–upper 95% CIs = −0.37 to −0.20) and was lower when individuals were more socially integrated (*β* = −0.21, lower–upper 95% CIs = −0.29 to −0.12). Moreover, the probability of using gestural sequences varied across species, being lower in orangutans (probability = 0.284, lower–upper 0.95 HPD = 0.251 to 0.317) than chimpanzees (probability = 0.381, lower–upper 0.95 HPD = 0.353 to 0.412) and siamangs (probability = 0.381, lower–upper 0.95 HPD = 0.350 to 0.410).

### (b) Dyadic relationship quality and gestural complexity

For M1-Dyad, the most complex model fit the data better than the simpler one (elpd difference: −13.2 ± 5.3). The dyadic repertoire size was higher overall when individuals had a stronger social bond (*β* = 0.14, lower–upper 95% CIs = 0.06 to 0.22) and it differed across species, being lower in chimpanzees (probability = 0.102, lower–upper 0.95 HPD = 0.074 to 0.132), intermediate in orangutans (probability = 0.168, lower–upper 0.95 HPD = 0.120 to 0.221) and highest in siamangs (probability = 0.271, lower–upper 0.95 HPD = 0.202 to 0.342). For M2-Dyad, M3-Dyad and M4-Dyad, the simpler models provided a better fit to the data than the more complex ones (elpd difference: −0.9 ± 4.4, −2.4 ± 1.1 and −6.6 ± 3.4, respectively), suggesting that the probability of accounting for recipients’ attentional states, flexibility and probability of using gestural sequences were not predicted by maternal kinship, dyadic bond or species.

### (c) The effectiveness of complex gestural use and social experience

For M5-Ind, the most complex model fit the data better than the intermediate and simplest ones (elpd difference: −25.4 ± 21.8 and −42.8 ± 24.5, respectively), suggesting that the probability of eliciting a response, at the individual level, increased with age (*β* = 0.17, lower-upper 95% CIs = 0.05 to 0.29) and when fewer gestural sequences were used (*β* = −2.30, lower–upper 95% CIs = −2.95 to −1.66) and it also differed across species, being lower in orangutans (probability = 0.603, lower–upper 0.95 HPD = 0.553 to 0.652), as compared to chimpanzees (probability = 0.676, lower–upper 0.95 HPD = 0.644 to 0.708) and siamangs (probability = 0.684, lower–upper 0.95 HPD = 0.652 to 0.715). Finally, for M5-Dyad, the most complex model fit the data better than the intermediate and simplest ones (elpd difference: −41.1 ± 14.0 and −37.0 ± 14.2, respectively), suggesting that the probability of eliciting a response, at the dyadic level, increased when both individuals were more likely to account for others' attentional states (*β* = 1.23, lower–upper 95% CIs = 0.66 to 1.81) and used fewer gestural sequences (*β* = −1.65, lower–upper 95% CIs = −2.19 to −1.11).

## 4. Discussion

Our study revealed important differences in gestural complexity depending on individual and dyadic measures of sociality. First, repertoire size was higher for dyads that had stronger social bonds (M1-Dyad). By spending more time together and more likely interacting across a variety of contexts, individuals with stronger social bonds may rely on a wider range of gesture types during social interactions. In our study, however, repertoire size was not predicted by individual indexes of sociality (M1-Ind). Repertoire size may strongly vary within species depending on the way in which gestures are operationalized. Here, we differentiated only between broad gesture types, not considering structural variants of one gesture type (cf. [16]). Whereas this approach has been repeatedly used in literature [25,29,38] and has allowed us to reach asymptote relatively quickly in all study groups (figure 1), finer-grained distinctions between gestural categories and/or bottom–up approaches to the study of gestures (e.g. assessing the multiple forms of gestures; see e.g. [54]) might reveal a much stronger role of individual sociality by highlighting the subtle forms in which gestures are performed by different individuals, rather than their general occurrence.

Second, the probability of producing gestures when recipients were attentive varied across individuals (M2-Ind), as older individuals were more likely than younger ones to produce gestures when recipients were attentive, especially in the visual modality. Moreover, individuals of all species generally accounted for recipients’ attentional states when producing gestures, but they did it significantly more in the visual modality (i.e. when needed). These results suggest that apes adjust to the recipients' attentional state when producing gestures and that they do that by also accounting for the different modality they use. These findings confirm previous studies suggesting that great apes preferentially gesture when facing the recipient in all modalities (e.g. [18]), but that they also typically discriminate between gesture modalities in their everyday interactions, especially accounting for the recipients’ attention when this is most needed (i.e. in the visual modality [3,14–16]). The inclusion of lesser apes in our study further shows that these abilities are not limited to the great apes, but that they are shared at least by all the ape species. Future studies will need to better disentangle to what extent these abilities imply a complex cognitive understanding of others' mental states, and/or more simply reflect social strategies acquired through trial-and-error learning and social experience. At the dyadic level, however, we found no evidence that sociality predicts the probability of producing gestures when recipients were attentive (M2-Dyad). These findings are in contrast with our predictions, but also with literature suggesting that intentional instances of communication might be less frequent when partners are strongly related and the outcome of their interactions is more predictable [22].

Third, in our study, we found no variation in the ability to flexibly adjust gesture use depending on the social context, either at the individual (M3-Ind) or at the dyadic level (M3-Dyad). At the individual level, our flexibility index ranged from 0.72 to 0.79 depending on the species (and from 0.81 to 0.83 if also including gestures that were observed just once) and was therefore similar to that reported in literature (in wild chimpanzees: 0.84 [16]). These indexes suggest that a high proportion of gestures in apes is produced in the dominant context. However, the way in which we operationalized flexibility, despite being commonly used in literature, is largely dependent on the amount of observations conducted for each gesture type, individual (or dyad) and context, so that the flexibility is likely to increase very slightly, but steadily, as observation effort increases. Moreover, flexibility has been operationalized very differently across studies (e.g. means–end dissociation across contexts [55]; flexible adjustment of the gesture type depending on the recipient's attentional state [25,56,57]). Therefore, it is possible that longer-term studies, other measures of flexibility and/or more precise measures of sociality (see below) might lead to different results, showing a link between sociality and individual and dyadic variation in gestural flexibility.

Fourth, gestural sequences were more likely to be used by younger individuals and socially less integrated ones (M4-Ind), although we found no differences at the dyadic level (M4-Dyad). These results suggest that gestural sequences are mostly used by inexperienced individuals, and are in line with other research describing gestural sequences as redundant repetitions of the same gesture types [28,29], which at least in some species emerge as a consequence/in contexts of high arousal (e.g. for Sumatran orangutans [32]), becoming less frequent as individuals get older [28] and being mostly used when single gestures fail to elicit a response (e.g. [24,29]). Overall, these findings suggest that most gestural sequences do not entail elements of gestural complexity, although more studies are needed to better differentiate among different types of gestural sequences and sequences consisting of different signal types (see e.g. [28,58–60]).

Overall, our study showed that individual indexes of sociality, rather than dyadic ones, may better explain variation in gestural complexity. Across dyads, for instance, relationship quality failed to predict flexibility, probability of accounting for recipients' attentional states and of eliciting their response. However, there are several reasons why we might have failed to find a clear link between gestural complexity and dyadic indexes of sociality. In our study, for example, we operationalized dyadic relationship quality based on maternal kinship and matrixes of spatial proximity. However, these two measures might fail to exhaustively capture the complexity of ape relationships. Primates, for instance, are known to reliably discriminate paternal kin, and they may preferentially affiliate with paternal half-sisters than non-kin (e.g. [61–63]). Moreover, the intensity of dyadic social relationships is often assessed with composite indexes, in which multiple affiliative measures (e.g. grooming, proximity) are combined into a single score to obtain a more comprehensive evaluation of relationship quality (see e.g. [40]). Having only used proximity measures, our study might have failed to properly capture relationship quality across our study dyads. Therefore, including better measures of social relationships and taking into account paternal relationships might provide different results. In our study, this was unfortunately not possible, as we could not determine paternity for the chimpanzee group and we did not have enough data to assess composite indexes for all study groups. Furthermore, our study only included captive individuals, but patterns of sociality (and thus associations with gestural communication) may be very different in free-ranging primates, as free-ranging individuals may have much more flexibility to maintain or choose proximity with preferred conspecifics. Indeed, several studies in the wild have found a link between dyadic measures of sociality and gestural communication (e.g. [64–66]).

The effectiveness of gestural communication varied across individuals and dyads. At the individual level, the probability of receiving a response was higher for older individuals, and for individuals using fewer sequences (M5-Ind). At the dyadic level, the probability of obtaining a response was higher when both individuals accounted for the recipients’ attention, and when they used fewer sequences, with no differences depending on their relationship quality (M5-Dyad). These findings confirm that the complexity and effectiveness of gestural communication in apes generally increase with age (e.g. [22]), as individuals become more likely to account for others' attentional states, reducing the use of gestural sequences and becoming more effective. In particular, interactional experience and exposure to others’ gestures during lifetime may provide individuals with the opportunity to gradually learn how to produce more effective gestures, which more likely elicit recipients' responses, without this necessarily implying that apes must have a complex cognitive understanding of these processes. These results are in line with the repertoire tuning hypothesis [28], according to which gestural repertoires are tuned by experience and individuals become more effective with increasing age. They also confirm previous findings on the link between age and the effectiveness of gestural communication in chimpanzees [28]. Moreover, these findings confirm that gestural sequences are unlikely to mirror communication complexity (see above): the use of sequences does not increase the effectiveness of communication but rather increases when recipients are not responsive (see e.g. [25,28,67,68]).

Finally, our study evidenced some differences across species. First, we found that dyadic repertoire size was largest in siamangs, and smallest in chimpanzees (M1-Dyad), showing that, in siamangs, dyads generally used a higher proportion of gestures that belonged to the species repertoire. At first sight, given that repertoire size is generally considered a measure of communication complexity (see above), these results would suggest that gestural complexity is higher in siamangs. However, these results may also suggest that, in orangutans and especially in chimpanzees, social interactions among group members are less uniform, and dyads are more selective with regard to the kind of specific social interactions, the context and thus the gestures used with different social partners. Therefore, dyadic repertoire size may be larger in siamangs simply because they live in stable small groups with strong bonds between the adult pair, where there may be less variation in terms of possible social interactions (see [38] for a discussion), and social experience may play a weaker role, even if just for pruning their gestural repertoires [14,28,69,70]. Second, although all species more likely accounted for others’ attentional states when using visual gestures, orangutans did that more (M2-Ind). Moreover, orangutans also used fewer gestural sequences than the other species (M4-Ind), although they were also the species with the lowest probability of eliciting recipients' response (M5-Ind). Whether these results imply cognitive differences across species, however, is a question that unfortunately cannot be answered with our current dataset. Across species, our analyses only had an exploratory function, given that our sample size included species that differ in several socio-ecological aspects potentially linked to complex communication. Some authors, for instance, have suggested that the degree of flexibility in signal production is at least partly determined by the social system [71], and may vary depending on the levels of fission–fusion dynamics [3,15,33] or dominance styles of the species (e.g. [34,35]). In the future, the inclusion of more species and groups will be essential to systematically test whether these specific socio-ecological characteristics, or others, can reliably predict interspecies differences in the complexity and effectiveness of gestural communication.

Overall, our study confirms the link between sociality and complex gestural use and suggests that more complex forms of communication, at least in terms of intentional use, may be more effective at achieving the goals of communication. Through age and social experience, apes likely adapt their communication to the contingencies they experience, they learn to account for others' attentional states and decrease the use of gestural sequences, learn which gestures are more effective and how to best elicit a response by recipients. Future work will need to better account for the large inter- and intra-individual variation in how gestures are exactly produced, and further disentangle the relative contribution of social and ecological experiences to the development of complex gestural communication.

## Data Availability

Data and code are available at the Open Science Framework: https://doi.org/10.17605/OSF.IO/VF5AT [72].
